# Structural basis of RNA recognition and dimerization by the STAR proteins T-STAR and Sam68

**DOI:** 10.1038/ncomms10355

**Published:** 2016-01-13

**Authors:** Mikael Feracci, Jaelle N. Foot, Sushma N. Grellscheid, Marina Danilenko, Ralf Stehle, Oksana Gonchar, Hyun-Seo Kang, Caroline Dalgliesh, N. Helge Meyer, Yilei Liu, Albert Lahat, Michael Sattler, Ian C. Eperon, David J. Elliott, Cyril Dominguez

**Affiliations:** 1 Department of Molecular and Cell Biology, University of Leicester, Henry Wellcome Building, Lancaster Road, Leicester LE1 9HN, UK; 2 Institute of Genetic Medicine, Newcastle University, Central Parkway, Newcastle NE1 3BZ, UK; 3 Center for Integrated Protein Science Munich at Biomolecular NMR Spectroscopy, Department Chemie, Technische Universität München, Lichtenbergstr. 4, DE-85747 Garching, Germany; 4 Institute of Structural Biology, Helmholtz Zentrum München, Ingolstädter Landstrasse 1, DE-85764 Oberschleißheim, Germany; 5 School of Biological and Biomedical Sciences, University of Durham, South Road, Durham DH1 3LE, UK

## Abstract

Sam68 and T-STAR are members of the STAR family of proteins that directly link signal transduction with post-transcriptional gene regulation. Sam68 controls the alternative splicing of many oncogenic proteins. T-STAR is a tissue-specific paralogue that regulates the alternative splicing of neuronal pre-mRNAs. STAR proteins differ from most splicing factors, in that they contain a single RNA-binding domain. Their specificity of RNA recognition is thought to arise from their property to homodimerize, but how dimerization influences their function remains unknown. Here, we establish at atomic resolution how T-STAR and Sam68 bind to RNA, revealing an unexpected mode of dimerization different from other members of the STAR family. We further demonstrate that this unique dimerization interface is crucial for their biological activity in splicing regulation, and suggest that the increased RNA affinity through dimer formation is a crucial parameter enabling these proteins to select their functional targets within the transcriptome.

## Abstract (web summary)

Sam68 and T-STAR are members of the STAR family of proteins, which regulate various aspects of RNA metabolism. Here, the authors reveal structural features required for alternative splicing regulation by these proteins.

## Graphical abstract

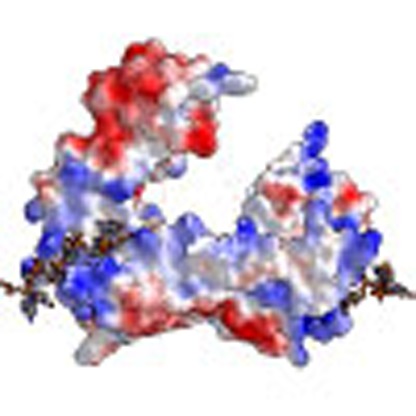

Sam68 (Src-associated protein in mitosis of 68 kDa)^1^,^2^ and T-STAR/SLM2 (testis-signal transduction and activation of RNA/Sam68-like mammalian protein 2)^3^,^4^ are members of the STAR family of proteins, composed of around 10 distinct proteins that are conserved through yeast, mammals and plants including the core splicing factor 1 (SF1)^5^. STAR proteins regulate various aspects of RNA metabolism, including pre-mRNA splicing, RNA export and stability and translation, and are highly regulated by signalling pathways. For example, Sam68 is phosphorylated by tyrosine kinases such as Src^1^,^2^ and serine/threonine kinases such as extracellular signal-regulated kinase 1 (Erk1) and cyclin-dependent kinase 1 (Cdk1) (refs 6, 7), arginine methylated by protein arginine methyltransferase 1 (PRMT1) (ref. 8), lysine acetylated by CREB-binding protein (CBP)^9^ and sumoylated^10^, and most of these modifications affect the functions of Sam68 in RNA metabolism, including its RNA-binding ability^9^,^11^,^12^,^13^, nuclear localization^8^ and effects on alternative splicing^6^,^14^. STAR proteins are therefore thought to provide a direct link between cell signalling and RNA metabolism.

Sam68 has been shown to have oncogenic properties^15^,^16^, and high expression of Sam68 correlates with poor prognosis in various cancers^17^,^18^. This is associated with the fact that Sam68 regulates the alternative splicing outcomes of CD44 (ref. 6), Bcl-x^14^, SRSF1 (ref. 19), cyclin D1 (ref. 20) and human papillomavirus 16 protein E6 (ref. 21), often favouring the production of the most oncogenic isoform. In addition, Sam68 is critical for controlling body mass index and thermogenesis through splicing of *mTOR*^22^, and controls nervous system functions through splicing of the *Neurexin* AS4 exons^23^. Furthermore, Sam68 plays a crucial role in human immunodeficiency virus (HIV) replication by assisting the nuclear export of unspliced and singly spliced HIV RNA^24^,^25^. T-STAR is a tissue-specific STAR protein mainly expressed in the testis and brain^3^,^4^,^26^, and regulates the alternative splicing of *CD44*, *Tra2β*, *Tau*, *VGEF* and *Neurexin* pre-mRNAs^26^,^27^,^28^. Similar to Sam68, T-STAR stimulates the activity of the HIV Rev protein^29^. The RNA-binding ability of T-STAR is regulated by tyrosine phosphorylation by Brk^30^ and arginine methylation by PRMT1 (ref. 13).

STAR proteins are defined by the presence of a highly conserved RNA-binding domain, the STAR domain, also responsible for homodimerization^31^, and composed of a central KH (K homology) domain flanked by two highly conserved regions, QUA1 and QUA2 (refs 5, 32). In terms of RNA-binding specificity, STAR proteins can be divided into two groups. The first group comprises the proteins SF1, QKI and GLD-1, which bind RNA motifs with a common (U/C)ACU(C/A)A(C/U) consensus sequence^33^,^34^,^35^. Structural studies of these proteins revealed that the QUA1 region is responsible for dimerization of the STAR domain^36^,^37^, while the KH domains recognize specifically the 3′ U(C/A)A(C/U) moiety of the RNA. The QUA2 regions play a central structural role by recognizing specifically the 5′ (U/C)AC moiety of the RNA^38^,^39^,^40^ and contacting both the QUA1 and the KH domains, stabilizing the overall orientation of the STAR dimer^39^. The second group of STAR proteins comprises the paralogues Sam68, T-STAR and SLM1. Originally, Sam68 was reported to bind poly(U) RNAs^1^. Later, systematic evolution of ligands by exponential enrichment (SELEX) experiments identified a shorter UAAA motif bound by Sam68 and a bipartite sequence containing a UAAA and a UUAA motifs bound by T-STAR and Sam68 (refs 41, 42). Structural studies have shown that the QUA1 domain plays a role in dimerization^43^, but the structural basis of RNA recognition and the mechanisms of action of the Sam68/T-STAR group of proteins in RNA metabolism remain unknown.

To gain mechanistic functional insights, we have deciphered the structural basis of dimerization and RNA recognition by Sam68 and T-STAR. We show that the dimerization of both T-STAR and Sam68 synergizes their binding affinity to target RNAs, and is essential for their function in splicing regulation *in vivo*. We speculate that homodimer formation may also contribute to splicing control of some pre-mRNAs through enabling looping out of regions of target RNAs.

## Results

### Unique mode of dimerization of T-STAR and Sam68 STAR domains

**Figure 1:**
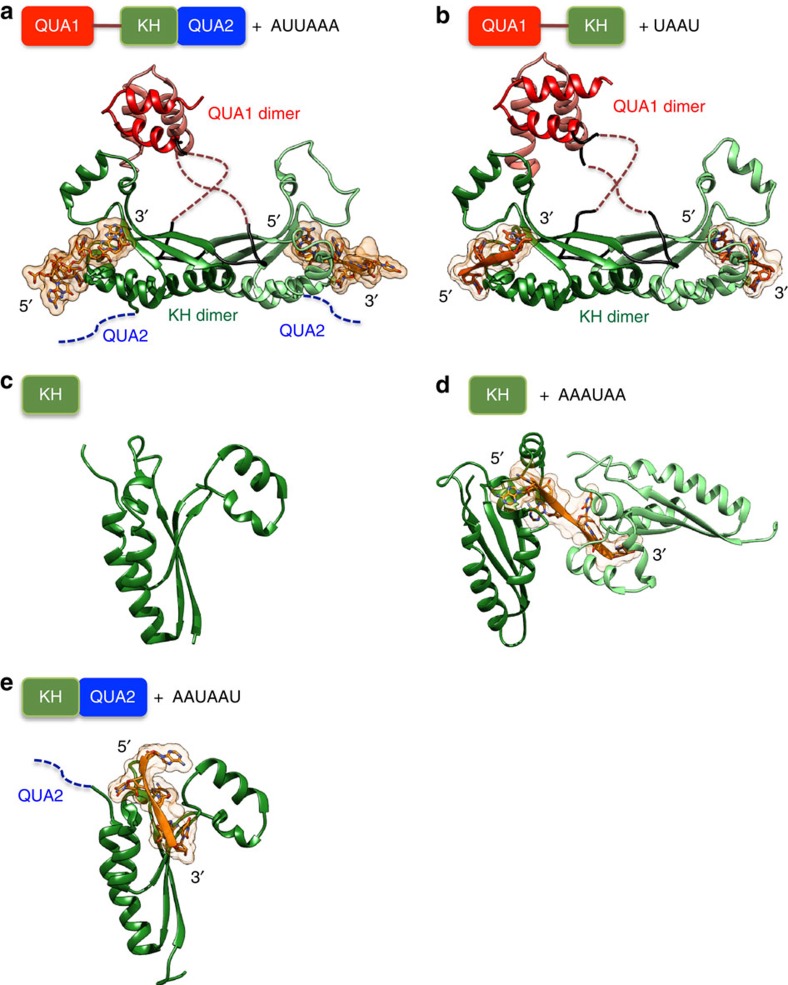
Crystal structures of T-STAR STAR, QUA1-KH, KH and KH-QUA2 domains, free and in complex with RNA. Overview of T-STAR STAR domain in complex with AUUAAA (**a**); QUA1-KH domain in complex with UAAU (**b**); KH domain free (**c**); KH domain in complex with AAAUAA (one KH bind the 5′ AAA moiety and another KH binds the 3′ UAA moiety) (**d**); and KH-QUA2 domain in complex with AAUAAU (**e**). The QUA1 dimer is in red and pink, the C-terminal half of the QUA1-KH linkers in black, the KH dimer in green and the RNA in orange. The QUA1 and KH dimers are labelled. The disordered N-terminal half of the QUA1-KH linker and the QUA2 domain are represented by brown and blue dashed lines, respectively.

We have shown previously using NMR spectroscopy that the isolated KH domains of Sam68 and T-STAR are sufficient for binding A/U-rich RNAs^44^. Here, we have determined the X-ray structures of the T-STAR KH domain in its free state, and in complex with AAAUAA; KH-QUA2 in complex with AAUAAU; QUA1-KH in complex with UAAU; and the full STAR domain in complex with AUUAAA (Fig. 1; Tables 1 and 2).

The structures of the T-STAR QUA1-KH and STAR domains in complex with RNA show that the QUA1 and the KH domains form compact dimers, with each KH domain binding one RNA molecule, while the QUA2 domain does not adopt a fixed orientation (Fig. 1a,b). The QUA1 domain adopts a helix-turn-helix motif involved in homodimerization, and its fold is very similar to the structure of isolated Sam68 QUA1 reported previously (backbone root mean square deviation (RMSD) of 0.61 Å)^43^. No electron density could be observed for the N-terminal half of the linker connecting the QUA1 to the KH domain (residues 35–42), suggesting that this region is disordered, while the C-terminal half of the linker (residues 43–53) is well defined in the structures. The KH domain of T-STAR adopts a classical type-I KH fold very similar to KH structures of other STAR proteins^38^,^39^,^40^. A comparison of free and RNA-bound structures of T-STAR show that the presence of the QUA1, QUA2 and the RNA do not induce any global structural changes of the KH domain (backbone RMSD ranging between 0.44 and 1.05 Å).

**Figure 2:**
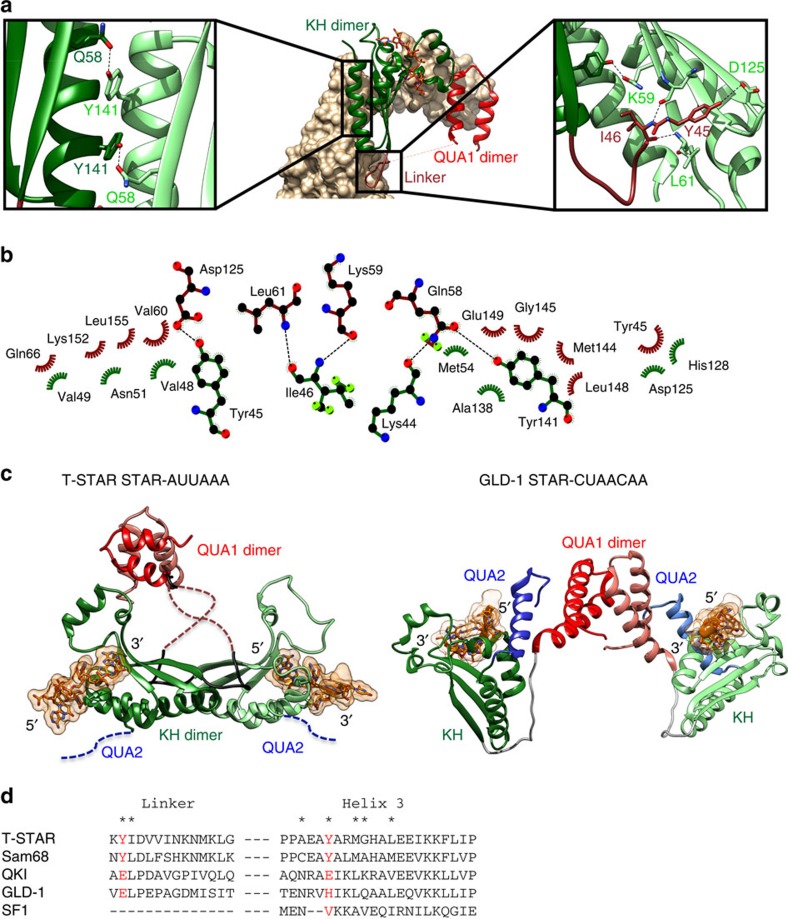
KH-linker dimer interface of the T-STAR STAR domain. (**a**) Centre: overview of the KH-linker dimerization interface of T-STAR STAR in complex with AUUAAA. One monomer is represented as ribbon and the other in surface representation. Left: close-up view of the specific intermolecular KH/KH interaction involving helix 3. Right: close-up view of the specific intermolecular contacts between the linker of one monomer and the KH domain of another monomer. (**b**) Summary of intermolecular contacts observed in the dimer formed by the KH domains and the QUA1-KH linkers. Figure was generated using the software LigPlot+^68^. (**c**) Comparison of the structures of T-STAR STAR domain in complex with AUUAAA and GLD-1 STAR domain in complex with CUAACAA (pdb:4JVY)^39^. (**d**) Sequence alignment of human T-STAR, Sam68, QKI, GLD-1 and SF1 displaying the linker region and α-helix 3 involved in T-STAR dimerization. Residues corresponding to T-STAR Y45 and Y141 are coloured red. Residues involved in the dimerization are marked with an asterisk.

Surprisingly, the KH domain and the C-terminal half of the QUA1-KH linker of T-STAR provide an additional dimerization interface (Fig. 2). This novel KH/linker interface covers 1,065 Å^2^ per monomer, almost twice as large as the QUA1 dimerization interface, and involves mainly the C-terminal α-helix 3 of the KH domain and the C-terminal half of the QUA1-KH linker. The KH interface is stabilized by a network of hydrophobic interactions involving residues A138, Y141, M144, G145 and L148, and an intermolecular hydrogen bond between Y141 in α-helix 3 and Q58 in β-strand 1 of the KH (Fig. 2a,b). The C-terminal half of the QUA1-KH linker (residues 43–53) also contributes to the dimer interface with the side chain of Y45 and the backbone of I46, forming a network of hydrogen bonds with the side chain of D125 and the backbone atoms of K59 and L61 (Fig. 2a,b). Interestingly, this dimer interface is very different from the dimer interface of other STAR proteins such as GLD-1 (Fig. 2c). This is consistent with the fact that all the residues of T-STAR involved in this novel interface (linker and α-helix 3) are conserved in Sam68 but different in QKI, GLD-1 and SF1 (Fig. 2d), and suggest that Sam68 and T-STAR have a similar dimerization interface, which is coherent with previous reports showing that T-STAR and Sam68 are able to heterodimerize^4^.

Strikingly, no electron density could be observed for the QUA2 region of T-STAR in all our data sets, suggesting that the QUA2 region does not adopt a well-defined orientation. Accordingly, the structure of T-STAR STAR in complex with AUUAAA overlay very well with the structure of T-STAR QUA1-KH in complex with UAAU with a backbone RMSD of 0.9 Å for the QUA1-KH atoms (Supplementary Fig. 1a).

**Figure 3:**
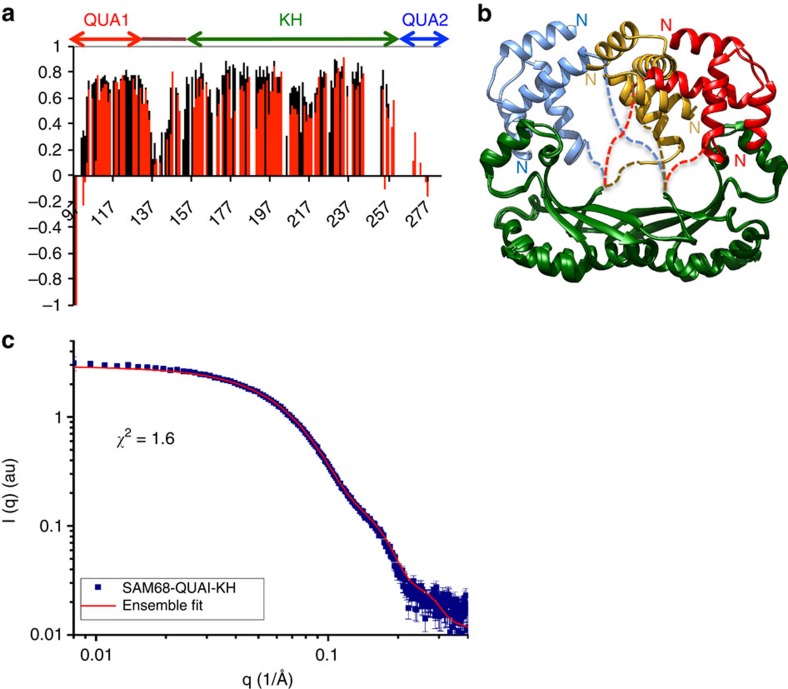
Structural characterization of Sam68 STAR domain in solution. (**a**) NMR {^1^H}-^15^N heteronuclear NOE of Sam68 QUA1-KH (black) and STAR domains (red). (**b**) Structural ensemble of Sam68 QUA1-KH derived from SAXS data analysis with EOM. Three structures are superimposed on the KH dimer (green) and the QUA1 domains are shown in red, gold and blue. The flexible linkers are represented by dashed lines. (**c**) Back-calculated data for the EOM structural ensemble (red) overlaid with the experimental SAXS data (blue).

T-STAR and Sam68 STAR domains display 70% amino acid sequence identity, have similar RNA sequence specificity^42^,^44^, and similar effects on alternative splicing of some pre-mRNAs^23^,^26^, suggesting that the structures of T-STAR and Sam68 STAR domains are similar, and that the QUA2 domain of Sam68 does not participate in the dimerization of the STAR domain. Consistent with this, the NMR ^1^H-^15^N correlation spectra of the Sam68 STAR and QUA1-KH domains in complex with AUUAAA RNA superimpose perfectly, demonstrating that the relative orientation of the Sam68 QUA1 and KH domains in solution is independent of the presence of the QUA2 region (Supplementary Fig. 1b) as observed in the crystal structures of T-STAR. Furthermore, {^1^H}-^15^N heteronuclear nuclear Overhauser effect (NOE) experiments of Sam68 QUA1-KH and STAR domains show that the QUA1 and KH domains and the C-terminal part of the linker adopt a rigid conformation (average {^1^H}-^15^N NOE values above 0.6), while the N-terminal half of the linker and the QUA2 region are flexible (average {^1^H}-^15^N NOE values below 0.3; Fig. 3a), in agreement with the structural features seen in T-STAR structures.

The crystal structures of T-STAR QUA1-KH and STAR domains show that the QUA1 dimer contacts only one KH domain (Fig. 1a,b). However, this is inconsistent with the presence of a single set of NMR chemical shifts that indicates a symmetrical dimer for the STAR domain in solution. To probe the relative orientations of the QUA1 and the KH domains in solution, we recorded small angle X-ray scattering (SAXS) data on T-STAR and Sam68 QUA1-KH and STAR domains in the absence and presence of an AAAUAA RNA (Supplementary Fig. 1c–e). The SAXS data confirm that both the T-STAR and Sam68 QUA1-KH and STAR domains are dimeric in solution (Supplementary Table 1). Interestingly, the presence of the QUA2 region in the STAR domains increases the radius of gyration and maximal dimensions both in the absence and presence of RNA (Supplementary Fig. 1d,e). This is consistent with the QUA2 domain being flexibly attached to the QUA1-KH domain dimer, and may explain that no electron density is observed for the QUA2 domain in the crystal structures of the T-STAR STAR and KH-QUA2 domains.

To analyze the solution conformation of the QUA1-KH dimer while taking into account that the linker connecting the QUA1 and KH domains is flexible (Fig. 3a), we performed ensemble calculations, using the ensemble optimization method (EOM) software^45^, of the Sam68 QUA1-KH module, since SAXS data for Sam68 were of better quality. We prepared a homology model of Sam68 QUA1-KH based on the crystal structure of T-STAR, and represented the QUA1 and KH dimers, as rigid bodies connected by a flexible linker of 11 residues for the EOM analysis. The accessible conformational space demonstrates that the QUA1 dimer samples multiple orientations relative to the KH dimer with a symmetric average position (Fig. 3b,c). As the SAXS data for T-STAR and Sam68 are qualitatively comparable (Supplementary Fig. 1d,e), similar structural arrangements are expected for the T-STAR and Sam68 QUA1-KH modules. From our NMR and SAXS data in solution, we conclude that the contacts between the QUA1 dimer and one KH domain observed in our X-ray structures are most probably due to crystal packing. Altogether, our data demonstrate that the STAR domains of Sam68 and T-STAR possess a novel dimer interface, different from the other members of the STAR family QKI and GLD-1.

### T-STAR and Sam68 recognize a short (A/U)AA RNA motif

**Figure 4:**
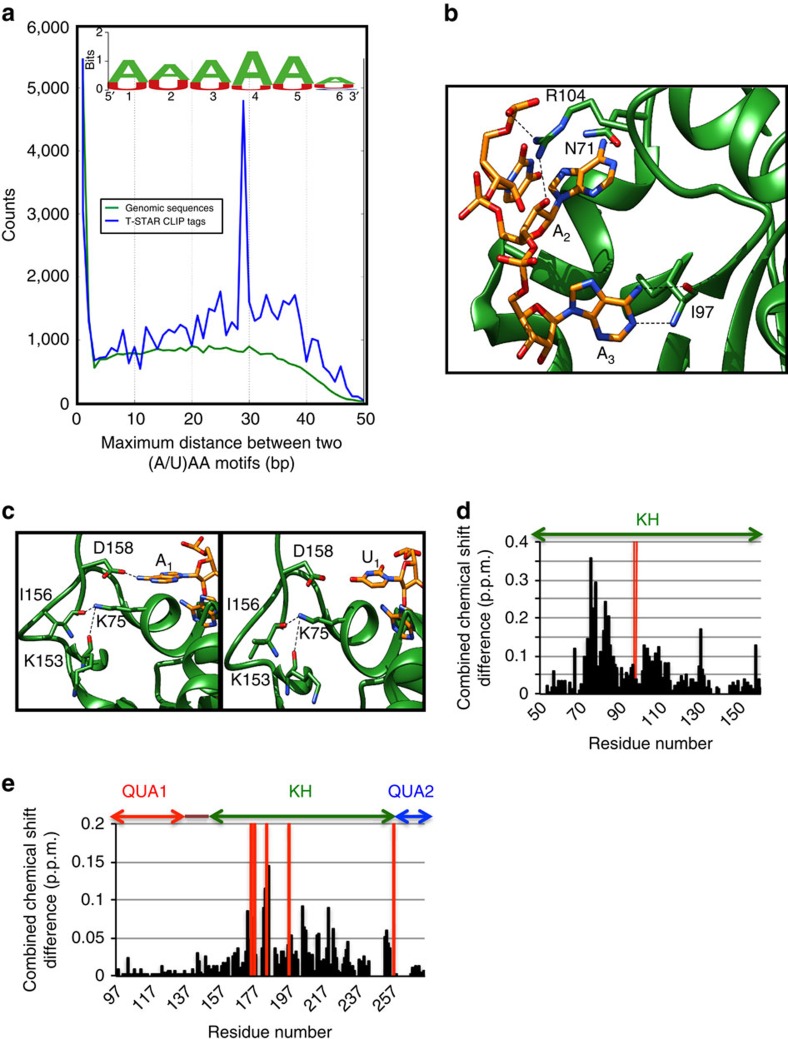
Structural basis of RNA recognition by T-STAR and Sam68. (**a**) Consensus binding site for T-STAR derived from alignment of full-length CLIP tags and maximum distance between two (A/U)AA sequences in each tag plotted against the normalized number of tags for T-STAR CLIP tags (blue), and random genomic region (green) as control. (**b**) Close-up view of the specific recognition of A_2_ and A_3_. (**c**) Close-up view of the specific recognition of A_1_ or U_1_. (**d**) NMR chemical shift perturbation upon addition of AAAUAA as a function of T-STAR KH amino acid sequence. I97 whose peak disappears upon complex formation is shown in red. (**e**) NMR chemical shift perturbation upon addition of AUUAAA as a function of Sam68 STAR amino acid sequence. Peaks that disappear upon complex formation are shown in red.

*In vitro* SELEX experiments previously identified a U(U/A)AA motif as high-affinity binding site for Sam68 and T-STAR^41^,^42^, and most RNA sequences bound by T-STAR displayed a bipartite nature containing a conserved UAAA and a UUAA motifs, separated by 3–25 nucleotides^42^. To investigate the RNA-binding motif *in vivo*, we performed a genome-wide high-throughput sequencing of RNA isolated by crosslinking immunoprecipitation (HITS-CLIP) experiment for T-STAR in adult mouse testis. In agreement with previous SELEX data, the CLIP tags are strongly enriched in adenine and uracil (Supplementary Table 2). However, the consensus motif derived from *in vivo* CLIP differs from the *in vitro* SELEX consensus motif. Our tags are highly enriched in adenines and suggest that T-STAR could bind preferentially poly-A RNA sequences (Fig. 4a). We next investigated whether HITS-CLIP data could also confirm the bipartite nature of the target RNA sequences, and identified an enrichment of sequences in which two (A/U)AA motifs are separated by a maximum distance of around 30 nucleotides (Fig. 4a).

All our structures of T-STAR in complex with RNA display a very similar mode of RNA recognition (Supplementary Fig. 2), and show that the KH domain specifically recognizes three nucleotides with the sequences U_1_A_2_A_3_ or A_1_A_2_A_3_ as illustrated for the structure of T-STAR KH-AAAUAA where one KH domain binds the 5′-AAA and another KH binds the 3′-UAA moieties of the RNA (Fig. 1d; Supplementary Fig. 2a). The RNA lies in the classical KH hydrophobic groove that comprises α-helices 1 and 2, β-strand 2, the GXXG loop and the variable loop^46^,^47^. The base of the nucleotide A_3_ is specifically recognized through intermolecular hydrogen bonds with the backbone atoms of I97 in β-strand 2 mimicking a Watson-Crick base pair (Fig. 4b). The base of the nucleotide A_2_ is specifically recognized through an intermolecular hydrogen bond to N71 side chain (Fig. 4b). Finally, the nucleotide A_1_ or U_1_ is stabilized by van der Walls contacts to G74, K75 and G78 of helix 1 (Fig. 4c), and A_1_ is stabilized by a hydrogen bond to the side chain of D158 at the C-terminus of the KH domain (Fig. 4c). Additional nucleotides located 5′ or 3′ of the (A/U)AA motif are visible in our structures but do not make specific contacts to the protein (Supplementary Fig. 2), suggesting that only the (A/U)AA motif is specifically recognized by T-STAR. All the KH-RNA contacts observed in the X-ray structures are consistent with NMR chemical shift perturbation experiments showing that the residues of T-STAR KH and Sam68 STAR that display the largest chemical shift perturbations upon RNA binding correspond to the residues that contact the RNA in the X-ray structures (Fig. 4d,e; Supplementary Fig. 3). To confirm the specificity of the interaction, we measured the affinity of T-STAR and Sam68 QUA1-KH for 5-mer RNAs derived from the Sam68 consensus A_1_U_2_A_3_A_4_A_5_ by fluorescence polarization (FP; Supplementary Table 3). These results are consistent with our structures and confirm that only an A at positions 3 and 4 of the 5mer is tolerated, while position 2 accommodates preferentially A or U, and the flanking residues at positions 1 and 5 are not specifically recognized, clearly defining the consensus RNA sequence for Sam68 and T-STAR recognition as N(A/U)AAN.

The absence of electron density for the QUA2 domain in our X-ray data sets suggests that the QUA2 domain of T-STAR does not contribute to RNA binding. This is consistent with NMR chemical shift perturbation experiments of Sam68 STAR domain with AUUAAA, where RNA binding does not induce any chemical shift perturbations for the residues in the QUA2 region (Fig. 4e). To further confirm that the QUA2 domain of T-STAR and Sam68 is not involved in RNA binding, we measured the affinity of T-STAR and Sam68 QUA1-KH and STAR domains for previously characterized high-affinity RNA sequences bound by Sam68 (G8.5 and G7.1)^41^, or T-STAR (SRE-4 (SLM2 response element 4; ref. 42) and *Neurexin2* (ref. 26)). In agreement with our NMR and X-ray data, the presence of the QUA2 region in both Sam68 and T-STAR constructs did not increase the affinity for these RNAs (Table 3). In contrast, the presence of the QUA2 region of Sam68 seems to slightly decrease its affinity for RNA. This is probably due to steric effects of the larger KH-QUA2 construct, since our SAXS and NMR data show that the QUA2 region of Sam68 remains highly flexible and does not interfere with RNA binding. Altogether, our data demonstrate that the QUA2 regions of Sam68 and T-STAR are not involved in RNA binding, in contrast to the other members of the STAR family SF1, QKI and GLD-1 (refs 38, 39, 40).

### The KH dimerization is necessary for alternative splicing

The orientation of Sam68 and T-STAR KH dimers positions two (A/U)AA RNA motifs in an anti-parallel manner on opposite sides of the protein dimer with a distance >50 Å between the 3′ end of one RNA and the 5′ end of the other (Supplementary Fig. 4). This suggests that, for both T-STAR and Sam68, the QUA1-KH dimer can only bind the same single-stranded RNA (ssRNA) if two (A/U)AA elements are separated by more than 15 nucleotides and is consistent with our HITS-CLIP data in which an enrichment of two (A/U)AA separated by 30 nucleotides is observed (Fig. 4a). We therefore measured the affinity of Sam68 and T-STAR for RNAs containing two UAAA-binding sites connected by linkers of 5, 10, 15, 20 and 30 cytosines, since poly-C does not bind T-STAR and Sam68 STAR domains (Supplementary Table 3). In agreement with our structural studies, while linkers of 5, 10 or 15 cytosines do not affect the affinity of the protein to the RNA, linkers of 20 and more cytosines induce an increase in affinity suggesting additive binding of T-STAR and Sam68 dimers to RNAs containing two binding sites distant by more than 15 nucleotides (Table 4). To confirm the role of the KH dimerization in this additivity, we mutated Y141 of T-STAR and Y241 of Sam68 into a glutamate, since QKI possess a glutamate at this position (Fig. 2d) and a negatively charged residue would interfere with the hydrophobic dimer interface. These mutants remain able to bind UAAA RNAs, indicating that the KH fold is not affected by the mutation. However, as expected, either the additivity of binding observed for T-STAR WT to longer RNAs is abolished by the mutation, or the additivity of binding occurs even for short linker sequences in the case of Sam68-Y241E (Table 4). This indicates that the structural integrity of the KH dimer plays a role in the affinity of the proteins to long RNAs, and allows for the definition of a more precise optimal bipartite (A/U)AA-N_>15_-(A/U)AA RNA sequence bound by Sam68 and T-STAR.

**Figure 5:**
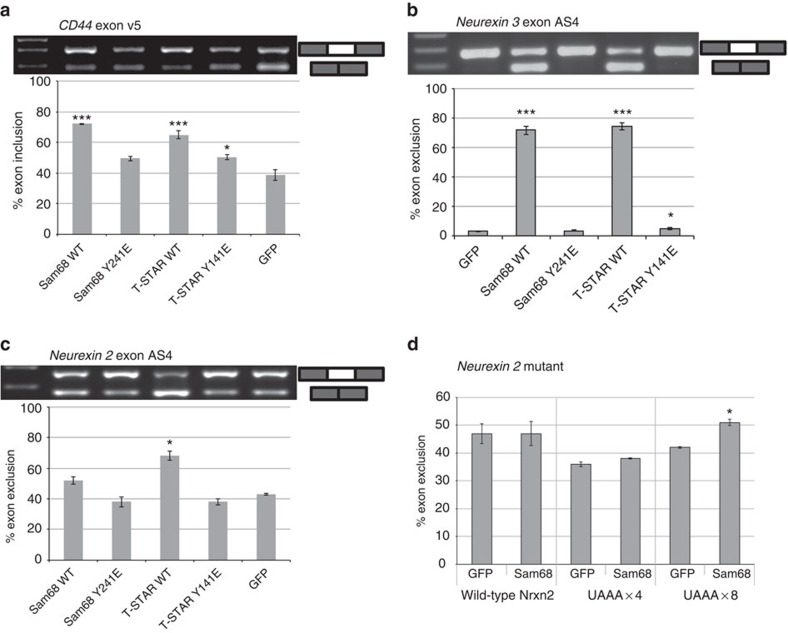
Structure–function relationship of Sam68 and T-STAR KH dimerization. (**a**–**c**) Effect of Sam68-Y241E and T-STAR-Y141E mutations on alternative splicing of *CD44* exon v5 (**a**), *Neurexin3* exon AS4 (**b**) and *Neurexin2* exon AS4 (**c**) minigenes. Top: agarose gel electrophoresis showing splicing of the minigenes in response to co-transfected proteins. Bottom: quantification of biological replicates from three independent co-transfection experiments. (**d**) Effect of Sam68 WT on a mutated *Neurexin*2 minigene before and after inclusion of a Sam68-binding site downstream of the exon AS4 5′ splice site. Bar charts were plotted in excel from at least three biological replicates and error bars represent the s.e.m. Statistical analyses were performed using GraphPad Prism (GraphPad software). *P* values were calculated using an independent two-sample *t*-test between GFP-transfected cells and Sam68- or T-STAR- (WT or mutant) transfected cells (statistical significance shown as: *0.01<*P*<0.05 and ***P<0.0001). Uncropped gels are shown in Supplementary Fig. 9.

To investigate the biological role of the KH dimerization for the function of T-STAR and Sam68 in alternative splicing, we co-transfected T-STAR-Y141E or Sam68-Y241E in HEK293 cells with *CD44*, *Neurexin2* or *Neurexin*3 minigenes. Sam68 and T-STAR have previously been shown to induce the inclusion of *CD44* exon v5 (refs 6, 27, 48), and the exclusion of *Neurexin*3 exon AS4 (ref. 26), while only T-STAR induces the exclusion of *Neurexin2* exon AS4 (ref. 26). T-STAR-Y141E and Sam68-Y241E localized predominantly in defined nuclear foci, a feature previously observed for wild-type Sam68 in cancer cells or Sam68 mutant proteins^49^ (Supplementary Fig. 5). In contrast to the wild-type proteins, the T-STAR-Y141E and Sam68-Y241E mutants failed to influence the alternative splicing of *CD44*, *Neurexin3* and *Neurexin2* (Fig. 5a–c), demonstrating that the KH dimerization interface is crucial for the function of these proteins in alternative splicing, both for activating (*CD44*) or repressing (*Neurexin2* and *Neurexin3*) exon inclusion. To also investigate the importance of the length of the RNA target site for efficient splicing, we used a mutated version of the *Neurexin2* minigene whose alternative splicing did not respond to Sam68 (ref. 26) and inserted either a (UAAA)_x4_ or a (UAAA)_x8_ sequence downstream of exon AS4. Consistent with a requirement for a bipartite sequence for functional activity as suggested by our structural data, the insertion of a long (UAAA)_x8_ sequence produced skipping of exon AS4 in response to transfection with Sam68, while insertion of the shorter (UAAA)_x4_ did not (Fig. 5d).

## Discussion

In this study, we provide the first high-resolution structures of T-STAR and Sam68, both in their free form and bound to their target RNAs. Our data indicate that these proteins function in splicing control as dimers, and this dimerization is mediated by a novel interface not found in the more distantly related STAR proteins quaking, GLD-1 and SF1. Taken together, our data reveal a unique mode of RNA recognition and dimerization by the STAR proteins Sam68 and T-STAR that is crucial for their function in alternative splicing. Importantly, we demonstrate that the QUA2 regions of T-STAR and Sam68 are neither involved in RNA binding nor in the dimerization. This is strikingly different from the other STAR proteins SF1, QKI and GLD-1 where the QUA2 domain plays a crucial role for both RNA binding and dimerization^38^,^39^,^40^ (Fig. 2c). Our data are, however, consistent with previous SELEX data showing that while the SF1/QKI/GLD-1 subfamilies specifically recognize a seven-nucleotide RNA sequence^33^,^34^,^35^, the consensus sequence for Sam68 and T-STAR is a smaller four-nucleotide A/U-rich motif^41^,^42^. Accordingly, single KH domains are well known to accommodate four nucleotides within their canonical binding groove^46^,^47^. Our data also show that Sam68 and T-STAR dimerization interfaces are very similar and mediated by the QUA1, the KH domains and the QUA1-KH linker but does not involve the QUA2 domain (Fig. 2). This is consistent with previous reports showing that the dimerization of Sam68 requires its QUA1 (ref. 43) but also its KH domain^31^, and that T-STAR and Sam68 are able to heterodimerize^4^. Dimers of KH domains have been previously reported from X-ray structures and solution NMR studies^46^,^47^,^50^, but differ significantly from the dimerization interface of T-STAR and Sam68. In most cases, KH dimerization interfaces involve an anti-parallel interaction of the β-strands 2, forming an extended six-stranded β-sheet^46^,^47^. Although helix 3 can sometimes stabilize the interaction, it does not form a network of hydrophobic contacts as observed for Sam68 and T-STAR. In contrast, β-strands 2 of T-STAR and Sam68 do not take part in the dimerization interface, due to the positioning of the QUA1-KH linker in between these strands. This is illustrated by a comparison of T-STAR KH dimer with poly-C binding protein 1 KH1 dimer (Supplementary Fig. 6)^51^. Therefore, the dimerization interface observed in the structures of T-STAR and Sam68 is to our knowledge novel and seems unique to T-STAR and Sam68 since T-STAR Y45 and Y141 are conserved in Sam68 (Y145 and Y241) but differ in QKI, GLD-1 and SF1 (Fig. 2d). These findings therefore define Sam68 and T-STAR as a novel subclass of STAR proteins, structurally distinct from GLD-1 and QKI.

Previous SELEX experiments identified a UAAA motif bound by Sam68, and a bipartite UAAA-N_3-25_-UUAA motif bound by T-STAR and Sam68 (refs 41, 42). Our CLIP and FP data go beyond this consensus sequence, and demonstrate that the optimal RNA sequence bound by T-STAR and Sam68 consists of a bipartite (A/U)AA-N_>15_-(A/U/AA) motif (Table 4), and that poly(A) has the strongest affinity for both proteins (Supplementary Table 3). This sequence is consistent with our structural data and with our genome-wide *in vivo* CLIP data on mouse testis, where all tags are highly enriched in adenine and uracil (Supplementary Table 2), the derived consensus motif is poly(A), and there is a strong enrichment for RNAs containing two (A/U)AA motifs separated by more than 20 nucleotides (Fig. 4a). Accordingly, natural pre-mRNA targets of Sam68 and T-STAR often contain bipartite RNA sequences with linker lengths >15 nucleotides or contain RNA sequences larger than 30 nucleotides that contain multiple (A/U)AA-binding motifs^6^,^22^,^23^,^26^,^48^,^52^.

It has previously been suggested that Sam68 binds preferentially UAAA motifs in loop regions of structured RNAs, and that the structural context of the RNA influences Sam68-RNA binding^52^. However, many natural target RNAs of Sam68 that have been characterized—such as CD44, neurexin1, mTOR or SRSF1—are not predicted to form secondary structures around the Sam68-binding sites and Mfold analysis of the 40-nucleotide long sequences obtained from *in vivo* genome-wide CLIP experiments did not identify any propensity for secondary structure formation surrounding the (A/U)AA motifs. Similarly, previous analysis of SELEX sequences bound by T-STAR did not identify any secondary structures^42^. This suggests that the regions near Sam68 and T-STAR-binding sites do not have a significant propensity to form secondary structures, and is consistent with previous structural studies of KH/RNA complexes showing that the KH is a *bona fide* ssRNA-binding domain.

Most RNA-binding domains, such as the RNA recognition motif (RRM) and the KH domains bind specifically only four to five nucleotides and therefore, most splicing factors contain multiple RNA-binding domains (RRM or KH) to increase both affinity and specificity to their pre-mRNA targets. The STAR family is rather unique, in that it only contains a single KH domain, and specificity and affinity is thought to arise from their ability to dimerize. Our structural data explain clearly how the unique dimerization interface of Sam68 and T-STAR contribute to both specificity and affinity to the RNA by recognizing the bipartite (A/U)AA-N_>15_-(A/U)AA RNA sequence. Interestingly, our data also indicate that the mutations in Sam68-Y241E and T-STAR-Y141E not only impair their dimerization and RNA-binding properties but also affect protein localization in the nucleus (Supplementary Fig. 5). Although the wild-type proteins display a predominantly diffuse nuclear localization, these mutants display a strong localization in nuclear foci termed Sam68 nuclear bodies (SNBs), which is consistent with earlier reports of other mutants of Sam68 (ref. 49). Notably, three sets of mutants that were shown previously to induce a localization of Sam68 to SNBs^49^ can now be explained in the light of our structural data. Two of these mutations (Sam68 R204C and Sam68 N171D/F172L) correspond to residues that are directly involved in RNA binding. The corresponding residues of T-STAR (R104 and N71) make specific hydrogen bonds to the RNA (Fig. 4b), and mutating these residues would impair RNA binding. The third mutation involves the deletion of Sam68 KH loop 1 (residues 164–171). The corresponding residues of T-STAR are at the dimer interface, and their deletion would certainly impair the dimerization (Fig. 2a,b). The subcellular distribution of these mutants indicates that interfering with either the dimerization or the RNA-binding activities of Sam68 and T-STAR induces a change in nuclear localization, and suggest that the localization of Sam68 to SNBs is a consequence of its failure to effectively bind its pre-mRNA targets.

The mechanisms by which Sam68 and T-STAR regulate alternative splicing remain poorly understood. Since the RNA sequence recognized by Sam68 could be found in numerous pre-mRNAs, how does Sam68 specifically affect only a subset of splicing events? Pre-mRNA molecules do not exist free in cells but interact with numerous RNA-binding proteins. Therefore, the consequence of Sam68-RNA interaction on splicing outcomes depends on the position of the bipartite RNA sequence relative to the splice sites, the competition for RNA binding by other splicing factors that compete for overlapping RNA sequences, the structure of the RNA or even post-translational modifications that are well known to affect Sam68 functions in alternative splicing. In some cases, it was proposed that Sam68 binding near splice sites can synergize or compete with the recruitment of other splicing factors, such as U2AF, hnRNP A1 or U1-70K^14^,^20^,^48^. The dimerization of the KH domain observed in our structures also suggests that Sam68 and T-STAR could regulate alternative splicing of some pre-mRNAs by bringing two distant UAA motifs into proximity and looping out regions of the pre-mRNA. Depending on the location of the binding sites, this could promote exon inclusion or skipping of alternative exons. Accordingly, sequence analysis of Sam68-dependent neuronal exons showed that Sam68-binding sites are enriched in the 200 nucleotides upstream and downstream of Sam68 target exons^53^. For example, it was previously shown that inclusion of SRSF1 exon 5 is stimulated by Sam68, and two functional Sam68-binding sites were identified in the upstream intron 4, one near the 5′ splice site and the other near the 3′ splice site^19^. Our structures suggest that binding of a Sam68 QUA1-KH dimer bring these two sites in close proximity, promoting exon 5 inclusion in the mature mRNA (Supplementary Fig. 7a). In contrast, Sam68 was shown to induce skipping of epsilon-sarcoglycan exon 8 and two intronic binding sites have been characterized, one located upstream and one downstream of the target exon^53^,^54^. In that case, binding of Sam68 dimer to these sites would promote the looping out and skipping of exon 8 (Supplementary Fig. 7b). Our structural data therefore suggest that Sam68 and T-STAR could influence splice site choices by bringing distant binding sites into close proximity. This mechanism of action would be similar to previously proposed models for other splicing factors such as PTB^55^, hnRNP A/B or hnRNP F/H^56^.

Finally, accumulating evidence suggests that Sam68 has oncogenic properties^15^,^16^, making it a potential therapeutic target. We show here that disruption of the KH dimer interface impairs Sam68 regulation of *CD44* and *Neurexin3* alternative splicing (Fig. 5a,b). Similarly, mutation of QUA1 residues disrupting the dimerization affected *CD44* alternative splicing^43^. Because the newly identified dimerization interface reported here is unique and specific to Sam68 and T-STAR, our structures provide an attractive template for designing specific drugs targeting the dimer interface and preventing the function of Sam68 in post-transcriptional gene regulation.

## Methods

### Protein and RNA production

Sam68 STAR (amino acids 97–283), KH-QUA2 (150–283) and KH (150–260) domains, and T-STAR STAR (1–183), KH-QUA2 (50–183) and KH (50–160) domains were cloned by the University of Leicester Cloning service (X. Wang, Protein Expression Laboratory (Protex), www2.le.ac.uk/departments/molcellbiol/facilities/protex) using the pLEICS-01 vector (Supplementary Table 4). All plasmid constructs were verified by DNA sequencing (PNACL, Leicester). Recombinant plasmids were transformed into Rosetta BL21 DE3 cells and expressed in 4 l of 2TY medium or M9 minimal medium supplemented with ^15^NH_4_Cl. At an optical density of 0.5, cultures were transferred to 20 °C for 1 h, and protein expression was induced with 400 μM isopropylthiogalactoside (IPTG) for 16 h at 20 °C. Proteins were purified by affinity chromatography using Ni-NTA agarose (Qiagen) followed by tobacco etch virus (TEV) cleavage during overnight dialysis in phosphate buffer (20 mM sodium phosphate (pH 7), 100 mM sodium chloride and 10 mM β-mercaptoethanol) at 4 °C. Because short ssRNA oligonucleotides are easily prone to degradation, 5 μl SUPERaseIN RNase inhibitor (Invitrogen) was added to the protein sample and further purified by size-exclusion chromatography on a Superdex 75 10/300 (GE Healthcare) into the desired buffer.

### Site-directed mutagenesis

Site-directed mutagenesis was carried out using overlap extension PCR with primers that contained the site of mutation centrally (Supplementary Table 4). Two PCR reactions were carried out. The products of these PCR reactions were purified and used as template for a second round of PCR using the 5′ and 3′ construct primers. This final PCR product was cloned by the University of Leicester Cloning service using the pLEICS-01 vector.

### X-ray crystallography

All the proteins constructs were dialyzed against 10 mM Tris (pH 7.0), 50 mM NaCl and all crystallization trials were performed using the sitting drop vapour diffusion method at 4 °C. The free KH domain and the KH-AAAUAA RNA crystallized as described previously^44^. The T-STAR QUA1-KH domain in complex with UAAU RNA crystallized in 0.1 M MIB (sodium malonate, imidazole, boric acid; pH 7.0) and 20% polyethylene glycol (PEG) 3350 at a protein concentration of 15–20 mg ml^−1^ and a protein/RNA molar ratio of 1:2. The T-STAR STAR domain in complex with AUUAAA RNA crystallized in 0.2 M NaCl, 0.1 M Na-HEPES (pH 7.5) and 24% PEG 4000 at a protein concentration of 15–20 mg ml^−1^ and a protein/RNA molar ratio of 1:2. The T-STAR KH-QUA2 domain in complex with AAUAAU RNA crystallized in 0.1 M imidazole (pH 8.0) and 8% PEG 8000 at a protein concentration of 10 mg ml^−1^ (protein/RNA molar ratio of 1:2).

Crystals were flash-frozen in mother liquor containing either 15% glycerol (QUA1-KH and STAR) or 15% 2-Methyl-2,4-pentanediol (MPD) (KH-QUA2).

All data sets were collected at the Diamond Light Source and processed using X-ray Detector Software (XDS)^57^. A single-wavelength anomalous dispersion (SAD) data set of the SeMet T-STAR KH domain was collected at a 0.9793 Å wavelength and 1.59 Å resolution. The space group was assigned to P12_1_1 with four proteins per asymmetric unit. The phase of the KH free domain of T-STAR was solved using AutoSol^58^ and AutoBuild^59^, and the model was rebuild using COOT^60^ and refined with REFMAC5 (ref. 61). A data set of T-STAR KH domain in complex with AAAUAA RNA was collected at a resolution of 2.87 Å. The space group was assigned to C222_1_ with six proteins and one RNA per asymmetric unit. The phase was solved by molecular replacement using the structure of the SeMet KH domain as a template and the program PHASER^62^. The model was rebuild using COOT^60^ and refined with PHENIX^63^. Processing through XTRIAGE suggested pseudo-merohedral twinning (twin fraction of 0.198 with the Britton analysis), and the twin operator 1/2*h+1/2*k, 3/2*h−1/2*k, −l was used during the final refinement. The density map shows that the RNA adopts two positions in this data set. In one, the 5′ AAA moiety binds one KH and the 3′ UAA moiety binds another KH, while in the other, the 5′ AA binds one KH and the 3′ UAAA binds another KH. A data set of T-STAR KH-QUA2 domain in complex with AAUAAA RNA was collected at a resolution of 2.3 Å. The space group was assigned to P2_1_2_1_2_1_ with two proteins and one RNA per asymmetric unit. The phase was solved by molecular replacement using the structure of the SeMet KH domain as a template and the program PHASER^62^. The model was reconstructed using COOT^60^ and refined with REFMAC5 (ref. 61). A data set of T-STAR QUA1-KH domain in complex with UAAU RNA was collected at a resolution of 2.13 Å. The space group was assigned to P12_1_1 with two proteins and two RNAs per asymmetric unit. A model of the QUA1 domain of T-STAR was built using the NMR structure of Sam68 QUA1 domain^43^. The phase was then solved by molecular replacement using the structures of the SeMet KH domain and the QUA1 model as templates and the program PHASER^62^. The model was rebuild using COOT^60^ and refined with PHENIX^63^. A data set of T-STAR STAR domain in complex with AUUAAA RNA was collected at a resolution of 3.02 Å. The space group was assigned to P12_1_1 with two proteins and two RNAs per asymmetric unit. The phase was solved by molecular replacement using the structure of the QUA1-KH domain as template and the program PHASER^62^. The model was rebuild using COOT^60^ and refined with PHENIX^63^. The atomic coordinates of the structures of T-STAR free and in complex with RNAs have been deposited to the Protein Data Bank with accession numbers 5EL3 (T-STAR KH free), 5ELR (T-STAR KH-QUA2/AAUAAU), 5ELS (T-STAR KH/AAAUAA), 5ELT (T-STAR QUA1-KH/UAAU) and 5EMO (T-STAR STAR/AUUAAA), and representative 2Fo-Fc density maps are displayed in Supplementary Fig. 8.

### NMR

NMR samples contained proteins at concentrations between 200 μM and 1 mM in 10 mM Tris (pH 7), 100 mM NaCl and 0.1% β-mercaptoethanol for Sam68 constructs, and 20 mM NaH_2_PO_4_ (pH 6.2) and 50 mM NaCl for T-STAR constructs. Almost complete backbone resonance assignment of T-STAR KH and Sam68 STAR was achieved by using two-dimensional (^15^N–^1^H)-heteronuclear single quantum coherence spectroscopy (HSQC), three-dimensional (3D) HNCA, 3D HNCACB, 3D CBCACONH and 3D (^15^N–^1^H)-nuclear Overhauser effect spectroscopy (NOESY) spectra recorded at 303 K. All spectra were analysed with Sparky (T. D. Goddard and D. G. Kneller, Sparky—NMR Assignment and Integration Software, www.cgl.ucsf.edu/home/sparky/, 2008). Backbone chemical shifts of Sam68 STAR and T-STAR KH have been deposited to the BioMagResBank with accession numbers 26700 and 26701, respectively.

### Homology modelling

Homology modelling of Sam68 QUA1-KH was carried out using the software Modeller^64^, by using the X-ray structure of T-STAR QUA1-KH and an optimized sequence alignment. The quality of the models was assessed using Procheck^65^.

### SAXS

SAXS experiments were performed on a Rigaku BioSAXS-1000 instrument with a HF007 microfocus generator equipped with a Cu-target at 40 kV and 30 mA. Transmissions were measured with a photodiode beamstop, Ag-behenate was used for q-calibration and beam centre determination. Measurements were performed in multiple 900-s frames checked for beam damage and averaged. Proteins constructs were measured at 25 °C with concentrations ranging between 1 and 20 mg ml^−1^ in the same buffers as those used for NMR. Protein–RNA complexes were measured at a concentration of 10 mg ml^−1^ protein and a protein/RNA molar ratio of 1:1. Circular averaging and background subtraction was done with the Rigaku SAXSLab software v 3.0.1r1. Molecular weights were calculated from the Porod volumes as described previously^66^. Ensembles were generated and analysed with the EOM program^45^. Although the complex has a two-fold symmetry, no symmetry was given to the EOM software as a constraint to test whether the resulting ensemble would be symmetric. *D*_max_, Porod volumes and distance distribution were calculated with GNOM, all part of the ATSAS package V 2.5.0-2 (ref. 66). The latter ones were normalized to a maximum of one.

### Fluorescence polarization

FP experiments were carried out in black 96-well plates with a 50-μl sample volume per well in 10 mM Tris (pH 7), 100 mM NaCl and 0.1% β-mercaptoethanol. Sam68 and T-STAR domains were serial diluted across the plate from 200 to 0 μM. Fluorescein-labelled RNA was then added at 0.2 μM final concentration. Plates were analysed using a PerkinElmer Victor X5 plate reader at excitation wavelength of 531 nm and emission at 595 nm.

### HITS-CLIP

HITS-CLIP was performed using a non-commercial affinity purified antibody raised against T-STAR^3^. Mouse testis was sheared in PBS and irradiated three times at 400 mJ cm^−2^ and a wavelength of 254 nm using the Stratagene Stratalinker. The lysate was treated with DNase and RNase, followed by immunoprecipitation with 80 μl T-STAR antibody, and 3′ linker ligation. RNA bound to T-STAR was separated by SDS–polyacrylamide gel electrophoresis and a thin band at the size of 70 kDa (T-STAR migrates at ∼55 kDa and the molecular weight of 50 nt RNA is about 15 kDa) was cut out, RNA was recovered, ligated with 5′ linker and reverse transcribed into cDNA, which were then sequenced on a Roche 454 GS-FLX platform. Reads were processed to remove sequencing linkers and barcodes, filtered to remove PCR duplicates and mapped to the mouse genome (Mm9) using Bowtie^67^, allowing for two mismatches. Of the 150,801 reads processed, 98,340 (65.20%) were successfully aligned according to the above parameters. K-mer analysis was carried out using custom-written Python scripts calculating the frequency of occurrence of each possible 6-mer sequence in the CLIP data set to identify sequences that were over-represented in the T-STAR CLIP data set compared to randomly selected mouse genomic sequences of the same size as the CLIP tags. Statistical significance was determined using a chi-squared test, and all top 15 motifs had a *P* value <0.05 for enrichment in T-STAR CLIP tags versus controls. The WebLogo was derived from tags containing the top 15 enriched k-mers using the online program WebLogo (http://weblogo.berkeley.edu/logo.cgi).

### Splicing and localization assays

Minigene splicing experiments were carried out in HEK293 cells transfected using lipofectamine 2000 (Invitrogen). RNA was extracted with Trizol (Invitrogen), and analysed using a one-step RT–PCR (PCR with reverse transcription) kit from Qiagen, both using the standard protocol. RT–PCR experiments used 100 ng of RNA in a 5-μl reaction using primers within the β-globin exons of pXJ41; PXJRTF (5′-GCTCCGGATCGATCCTGAGAACT-3′) and PXJB (5′-GCTGCAATAAACAAGTTCTGCT-3′). Reactions were analysed by agarose gel electrophoresis and quantified by capillary gel electrophoresis. For localization assays, HEK293 cells were fixed after 24 h using paraformaldehyde, mounted in VECTASHIELD with 4,6-diamidino-2-phenylindole (DAPI) and then directly visualized for green fluorescence protein (GFP) expression using fluorescence microscopy.

## Additional information

**How to cite this article:** Feracci, M. *et al*. Structural basis of RNA recognition and dimerization by the STAR proteins T-STAR and Sam68. *Nat. Commun.* 7:10355 doi: 10.1038/ncomms10355 (2016).

## Supplementary Material

Supplementary Information Supplementary Figures 1-9 and Supplementary Tables 1-4.

## Figures and Tables

**Table 1:** Data collection, phasing and refinement statistics for SAD (SeMet) structures of T-STAR KH free.

	**Native**	**SeMet**
*Data collection*		
Beamline	I04-1	I04-1
Space group	P1 21 1	P1 21 1
*Cell dimensions*		
*a*, *b*, *c* (Å)	57.12, 85.92, 59.07	57.12, 85.92, 59.07
*α*, *β*, *γ* (°)	90, 117.23, 90	90, 117.23, 90
		
		*Peak*
Wavelength		0.9793
Resolution (Å)	50.79–1.592 (1.649–1.592)	50.79–1.592 (1.649–1.592)
*R*_merge_	0.08616 (0.6158)	0.08614 (0.6158)
CC1/2	0.999 (0.917)	0.999 (0.917)
CC*	1 (0.978)	1 (0.978)
*I*/*σI*	27.78 (4.06)	27.78 (4.06)
Completeness	1.00 (1.00)	1.00 (1.00)
Redundancy	13.7 (13.6)	13.7 (13.6)
Wilson B-factor	18.59	18.59
		
*Refinement*		
Resolution (Å)	50.79–1.592 (1.649–1.592)	
No. of reflections		
Total	924,651 (91,187)	
Unique	67,707 (6,705)	
*R*_work_/*R*_free_	0.1724–0.2050 (0.2215–0.2447)	
No. of atoms	4,256	
Protein	3,619	
Ligand/ion	5	
Water	632	
B-factors	29.12	
Protein	28.37	
Ligand/ion	24.63	
Water	33.41	
Root mean squared deviations		
Bond lengths (Å)	0.017	
Bond angles (°)	1.89	
Ramachandran plot		
% Favoured	99	
% Outliers	0.23	
Molprobity		
Clashcores	3.65	

**Table 2:** Data collection and refinement statistics for molecular replacement.

	**QUA1-KH**–**UAAU**	**STAR**–**AUUAAA**	**KH-QUA2**–**AAUAAU**	**KH**–**AAAUAA**
*Data collection*				
Beamline	Diamond I03	Diamond I03	Diamond I04-1	Diamond I04-1
Space group	P 1 21 1	P 1 21 1	P 21 21 21	C 2 2 21
*Cell dimensions*				
*a*, *b*, *c* (Å)	54.88, 46.06, 83.98	51.601, 79.967, 53.831	42.38, 45.56, 151.98	93.73, 162.22, 113.04
*α*, *β*, *γ* (°)	90, 96.36, 90	90, 101.05, 90	90, 90, 90	90, 90, 90
Resolution (Å)	43.5–2.13 (2.206–2.13)	25.68–3.03 (3.138–3.03)	43.64–2.3 (2.382–2.3)	81.16–2.87 (3.02–2.87)
*R*_merge_	0.05636 (0.6557)	0.08798 (0.3612)	0.08045 (0.8574)	0.1907 (0.7811)
CC1/2	0.999 (0.849)	0.988 (0.8)	0.998 (0.898)	0.989 (0.7)
CC*	1 (0.958)	0.997 (0.939)	1 (0.973)	0.997 (0.908)
*I*/*σI*	18.29 (3.26)	8.10 (1.96)	21.25 (3.32)	8.07 (2.53)
Completeness (%)	0.99 (1.00)	0.94 (0.93)	1.00 (1.00)	99.83 (99.03)
Redundancy	6.5 (6.6)	2.6 (2.6)	12.6 (12.9)	6.9 (7.2)
Wilson B-factor	47.40	55.64	46.13	49.63
Twin fraction	—	—	—	0.198
				
*Refinement*				
Resolution (Å)	43.5–2.13 (2.206–2.13)	28.51–3.03 (3.138–3.03)	43.64–2.3 (2.382–2.3)	81.16–2.87 (3.02–2.87)
No. of reflections				
Total	153,246 (15,486)	20,330 (1,967)	173,701 (17,282)	137,428 (14,177)
Unique	23,555 (2,352)	7,888 (771)	13,734 (1,340)	20,040 (1,962)
*R*_work_/*R*_free_	0.2043–0.2565 (0.2815–0.3532)	0.2207–0.2693 (0.2894–0.3744)	0.2273–0.2694 (0.2641–0.3884)	0.1875–0.2358 (0.2583–0.3236)
No. of atoms	2,597	2,606	1,927	5,631
Protein	2,418	2,350	1,800	5,356
RNA	168	256	106	260
Ion	0	0	0	15
Water	11	0	21	0
B-factors	66.46	55.88	56.71	52.05
Protein	66.31	55.93	56.60	52.62
RNA	69.98	55.39	60.44	39.89
Ion	—	—	—	46.08
Water	45.43	—	47.48	—
Root mean squared deviations				
Bond lengths (Å)	0.015	0.003	0.015	0.013
Bond angles (°)	1.51	0.669	1.81	1.46
Ramachandran plot				
% Favoured	96	98	98	95
% Outliers	0.34	0.35	0	1.5
Molprobity				
Clashcores	14.83	5.04	3.91	20.6

**Table 3:** Dissociation constants of T-STAR and Sam68 STAR and QUA1-KH domains to SELEX-derived AU-rich RNAs determined by fluorescence polarization.

**Kd (μM)**	**T-STAR**		**Sam68**	
	**STAR**	**QUA1-KH**	**STAR**	**QUA1-KH**
G8.5	11.7	8.1	36.1	10.3
G7.1	5.4	4.5	8.2	4.3
SRE-4	8.5	9.4	65.7	19.1
Nrxn2	1.1	1.7	3.7	2.2

G8.5: CUGGGUGACACACUAGCUAUAGCAUUAAAAGACCGAGCAAGU.

G7.1: UCCGGAUUGGCCUAAAUAGAUGCGCGAUAAUAAUAGAGUA.

SRE-4: UUUGGGGGUUCAAUAAAAAUUUUCACUAUCCUAUUAACAGUUCCGCCGCUCC.

Nrxn2: CCCAAUUAACUAACUAACUAACUUUAAAA.

**Table 4:** Dissociation constants of T-STAR QUA1-KH and STAR domains and Sam68 QUA1-KH wild type (WT) and Y141E or Y241E mutants to RNAs containing two UAAA-binding sites separated by a poly-C linker.

**Kd (μM)**	**T-STAR**				**Sam68**	
	**STAR**		**QUA1-KH**		**QUA1-KH**	
	**WT**	**Y141E**	**WT**	**Y141E**	**WT**	**Y241E**
UAAACCC	7.0	6.8	7.9	10.9	50.0	27.1
UAAA-C5-UAAA	5.9	5.1	5.5	5.3	42.7	4.6
UAAA-C10-UAAA	7.0	4.9	8.6	4.4	37.3	3.9
UAAA-C15-UAAA	6.7	4.7	6.8	4.7	26.3	4.0
UAAA-C20-UAAA	2.1	5.7	2.4	5.1	8.4	4.8
UAAA-C25-UAAA	1.4	5.2	1.8	6.2	4.0	5.5
UAAA-C30-UAAA	1.7	6.7	2.0	6.0	5.0	7.5
