# Supplementary material for: Structural basis of RNA recognition and dimerization by the STAR proteins T-STAR and Sam68
Source: Nat Commun. 2016 Jan 13;7:10355. doi: 10.1038/ncomms10355 (PMC4735526; doi:10.1038/ncomms10355)
Supplement: Supplementary Information — Supplementary Figures 1-9 and Supplementary Tables 1-4. [file ncomms10355-s1.pdf]

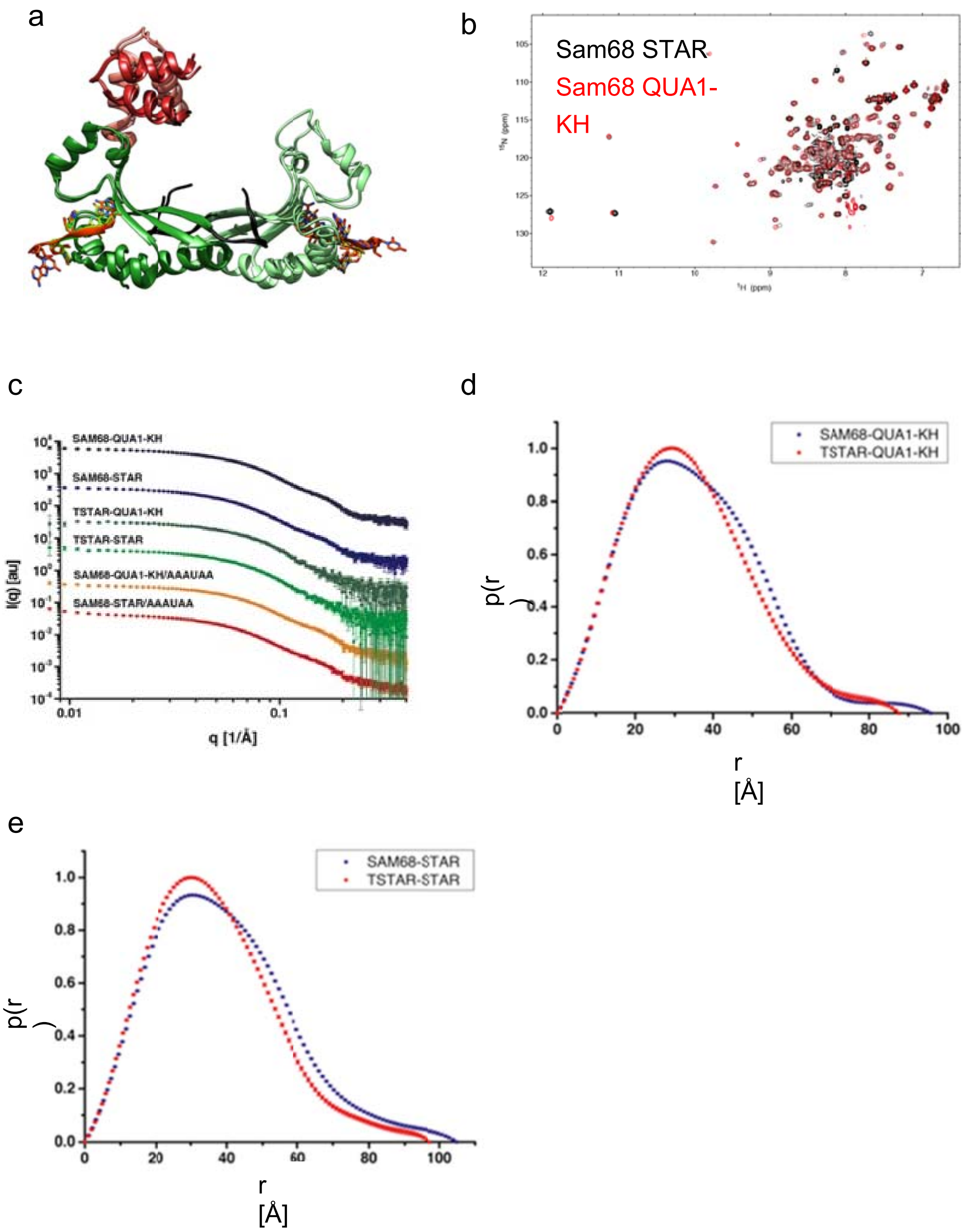


**Supplementary Figure 1: The QUA2 domain is not involved in the overall conformation of the STAR domain**

(a) Overlay of T-STAR QUA1-KH in complex with UAAU and T-STAR STAR in complex with AUUAAA structures. (b) HSQC spectra of Sam68 STAR (black) and QUA1-KH (red) domains in complex with AUUAAA. (c) Experimental SAXS data for the T-STAR and Sam68 QUA1-KH and STAR domains free and in complex with AAAUAA RNA. (d,e) Comparison of distance distributions of SAM68 and TSTAR QUA1-KH domains (d) and of SAM68 and TSTAR STAR domains (e). The maximum of both curves is at the same distance. TSTAR constructs are slightly more compact than Sam68 constructs.

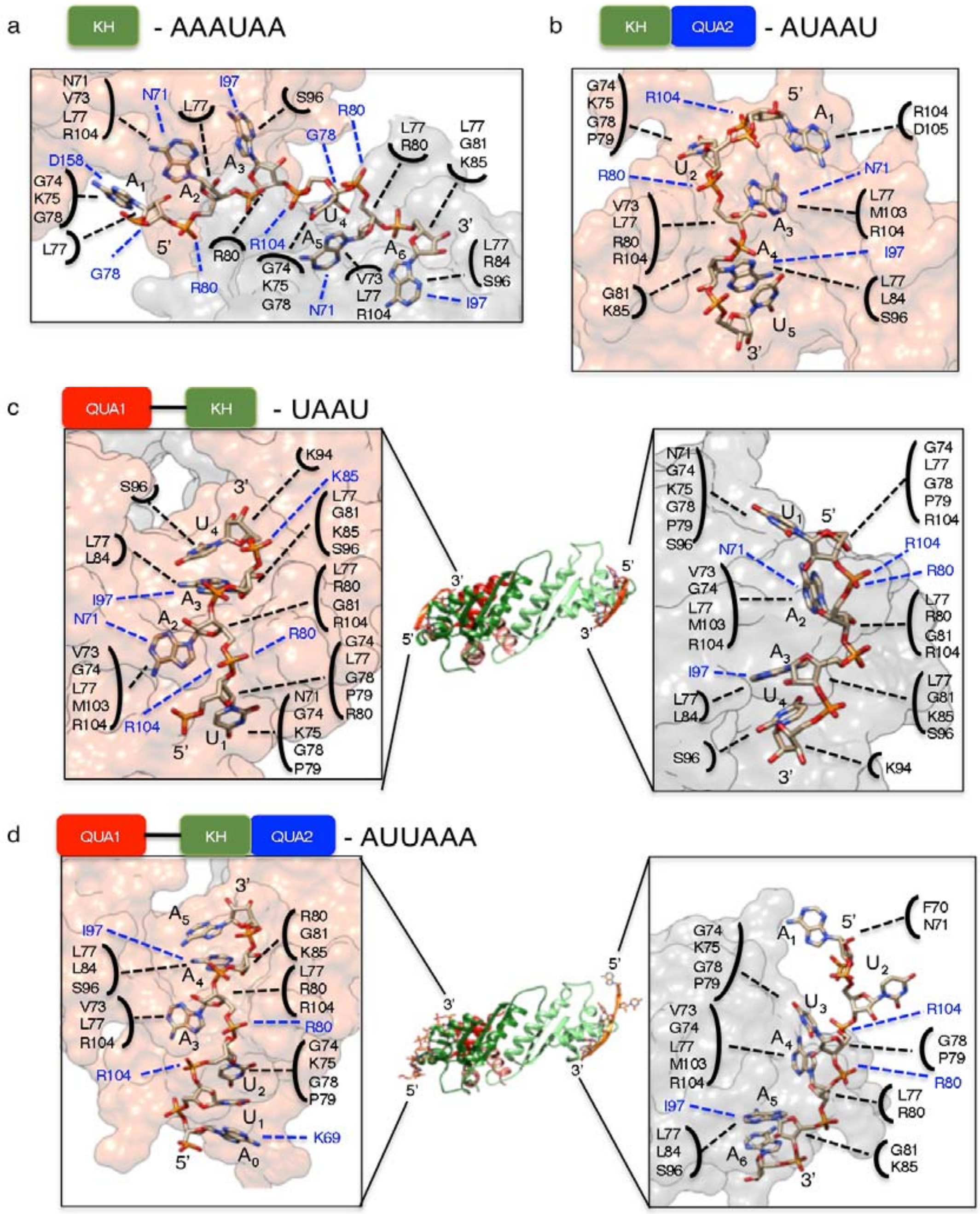


**Supplementary Figure 2: Protein-RNA contacts observed in the X-ray structures of T-STAR-RNA complexes.**

Intermolecular contacts between T-STAR KH and AAAUAA (the 5´ AAA motif is recognized by one KH and the 3´ UAA motif by another KH) (a), KH-QUA2 and AAUAAU (b**)**, QUA1-KH and UAAU (c), and STAR and AUUAAA (d). Black and blue lines indicate van der Waals contacts and hydrogen bonds, respectively.

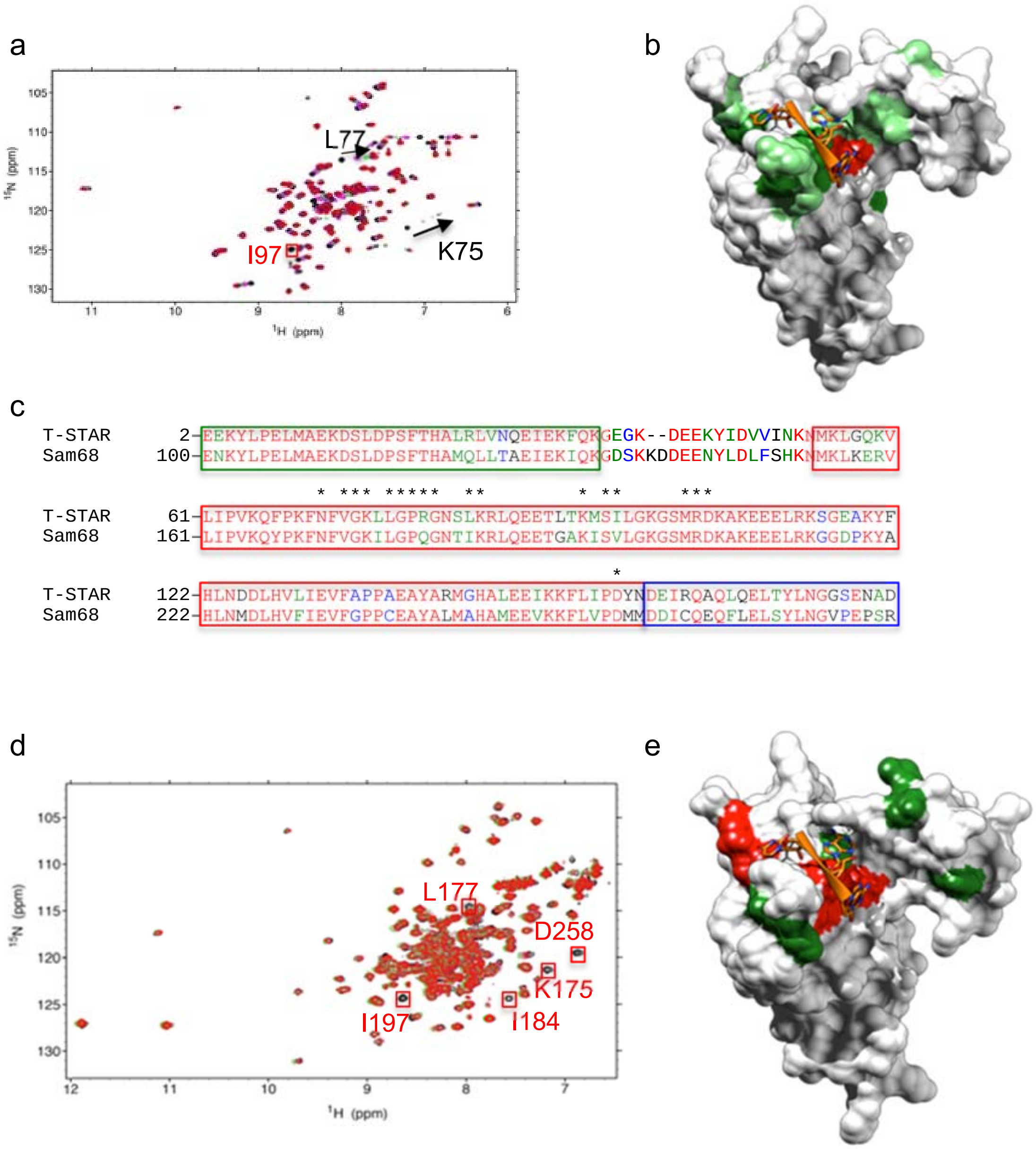


**Supplementary Figure 3: NMR derived RNA recognition by T-STAR KH domain and Sam68 STAR domain.**

(a) HSQC spectra of T-STAR KH free (black) and in complex with AAAUAA at protein:RNA molar ratios of 1:0.25 (magenta), 1:0.5 (green), 1:1 (blue) and 1:1.5 (red). (b) Amino acids that display a significant chemical shift perturbation or disappear upon RNA binding are colored on the surface representation of T-STAR KH-UAA structure. (c) Sequence alignment of T-STAR and Sam68 STAR domains. Amino acids corresponding to the QUA1, KH and QUA2 domains are boxed in green, red and blue, respectively. Amino acids that contact the RNA in the T-STAR-AAAUAA structure are marked with an asterisk. (d) HSQC spectra of Sam68 STAR free (black) and in complex with AUUAAA at protein:RNA molar ratios of 1:0.5 (magenta), 1:1 (green) and 1:1.2 (red). (e) Amino acids that display a significant chemical shift perturbation or disappear upon RNA binding are colored on the surface representation of Sam68 KH structural model, derived from T-STAR KH structure. The UAA RNA is positioned based on T-STAR KH-AAAUAA structure.

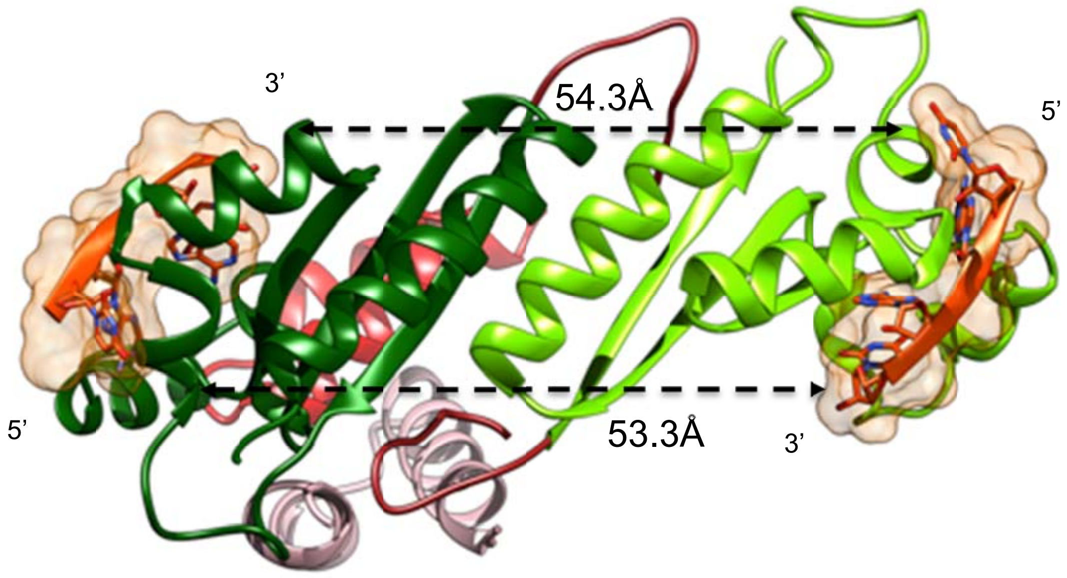


**Supplementary Figure 4: The KH dimerization brings two RNA binding elements on opposite sides of the dimer.**

Overview of T-STAR QUA1-KH structure in complex with UAAU. The distance between the 3´-end of one RNA and the 5´-end of the other is indicated.

GFP-Sam68 WT

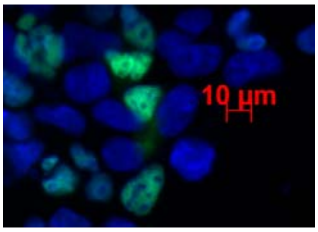


GFP-Sam68 Y241E

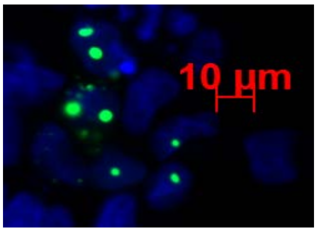


GFP-T-STAR WT

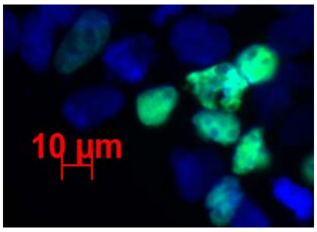


GFP-T-STAR Y141E

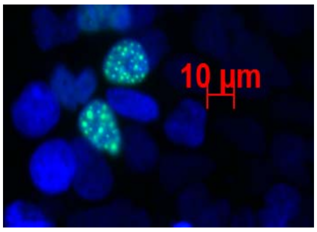


**Supplementary Figure 5: Localization of GFP-Sam68 WT and Y241E, and GFP-T-STAR WT and Y141E, in HEK293 cells.**

GFP-fusion constructs were transfected into HEK293 cells, and 24 h after transfection the protein expression patterns were analyzed by fluorescence microscopy. The cell nuclei were stained with DAPI.

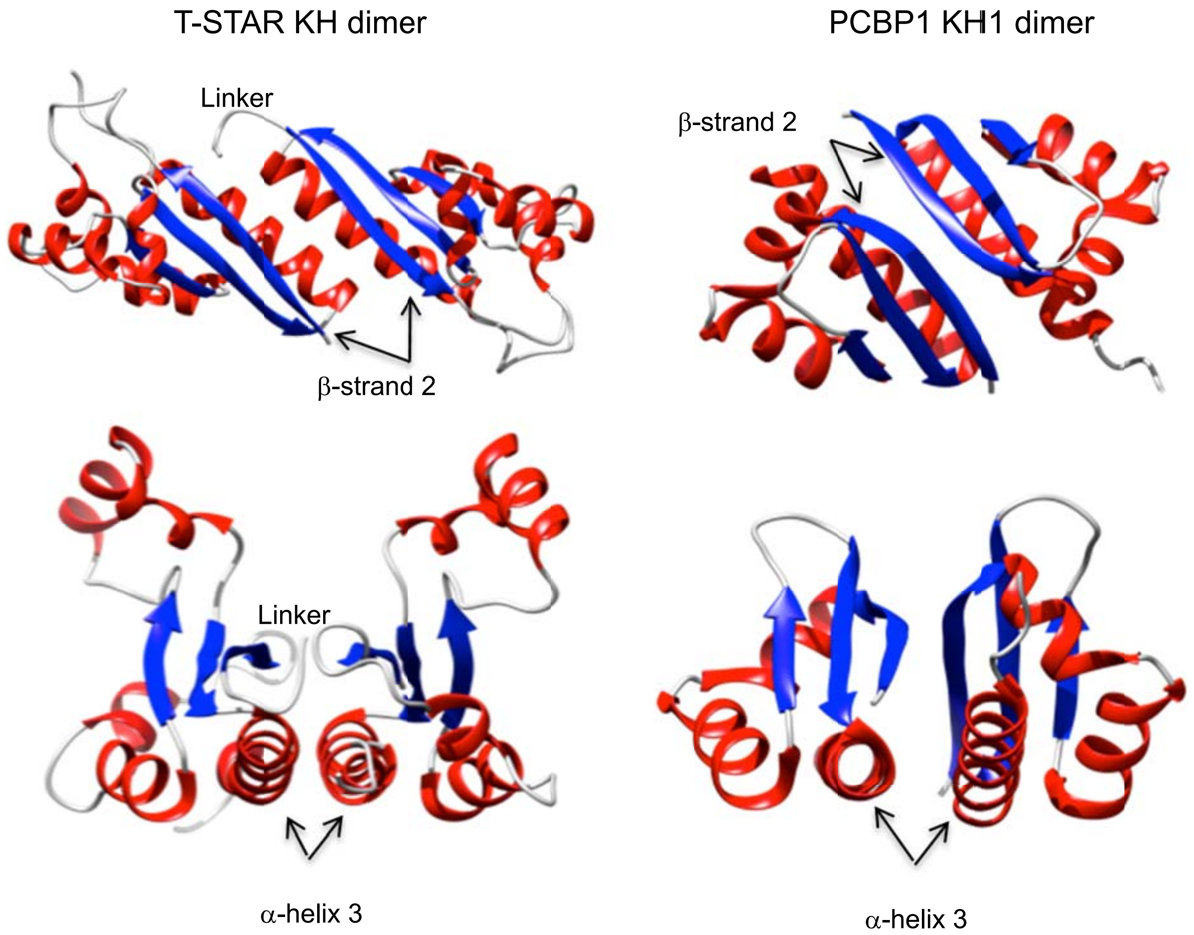


**Supplementary Figure 6: Comparison of T-STAR KH and PCBP1 KH1 dimerization interfaces.**

Top, β-sheet view of the structures. The dimer interface involves β-strands 2 of PCBP1 (right) but not of T-STAR (left). Bottom, α-helix 3 view of the structure. The dimer interface involves a large hydrophobic interface of α-helix 3 of T-TSTAR (left) but not PCBP1 (right).

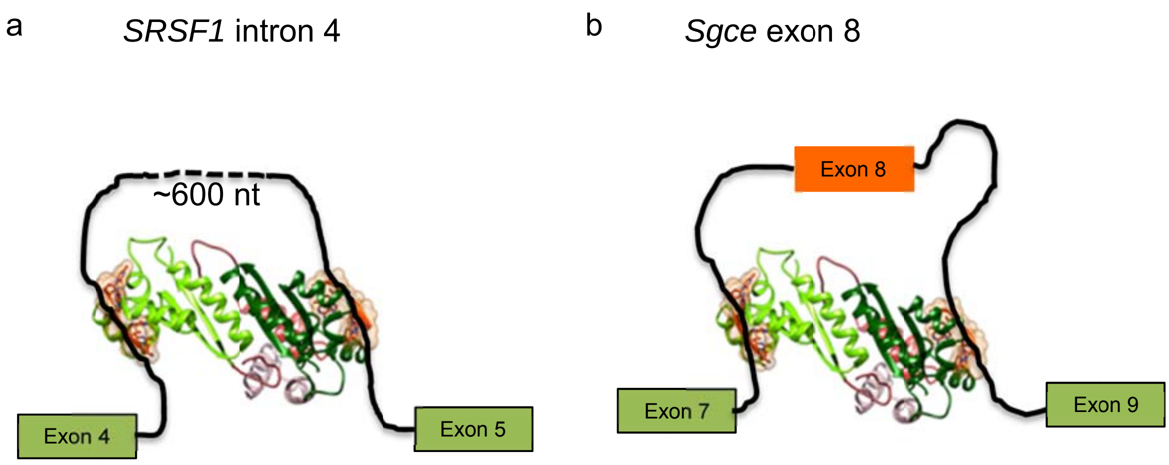


**Supplementary Figure 7: Sam68 could regulate splicing by looping out regions of the pre-mRNA.**

Structural models of Sam68 interaction with *SRSF1* (a), and *Sgce* (b) pre-mRNAs suggesting that Sam68 might function in alternative splicing control by looping out regions of the pre-mRNA, to promote exon inclusion or exon skipping, respectively.

T-STAR
STAR / AUUAAA

T-STAR
QUA1-KH / UAAU

T-STAR
KH

T-STAR
KH / AAAUAA

T-STAR
KH-QUA2 / AAUAAU

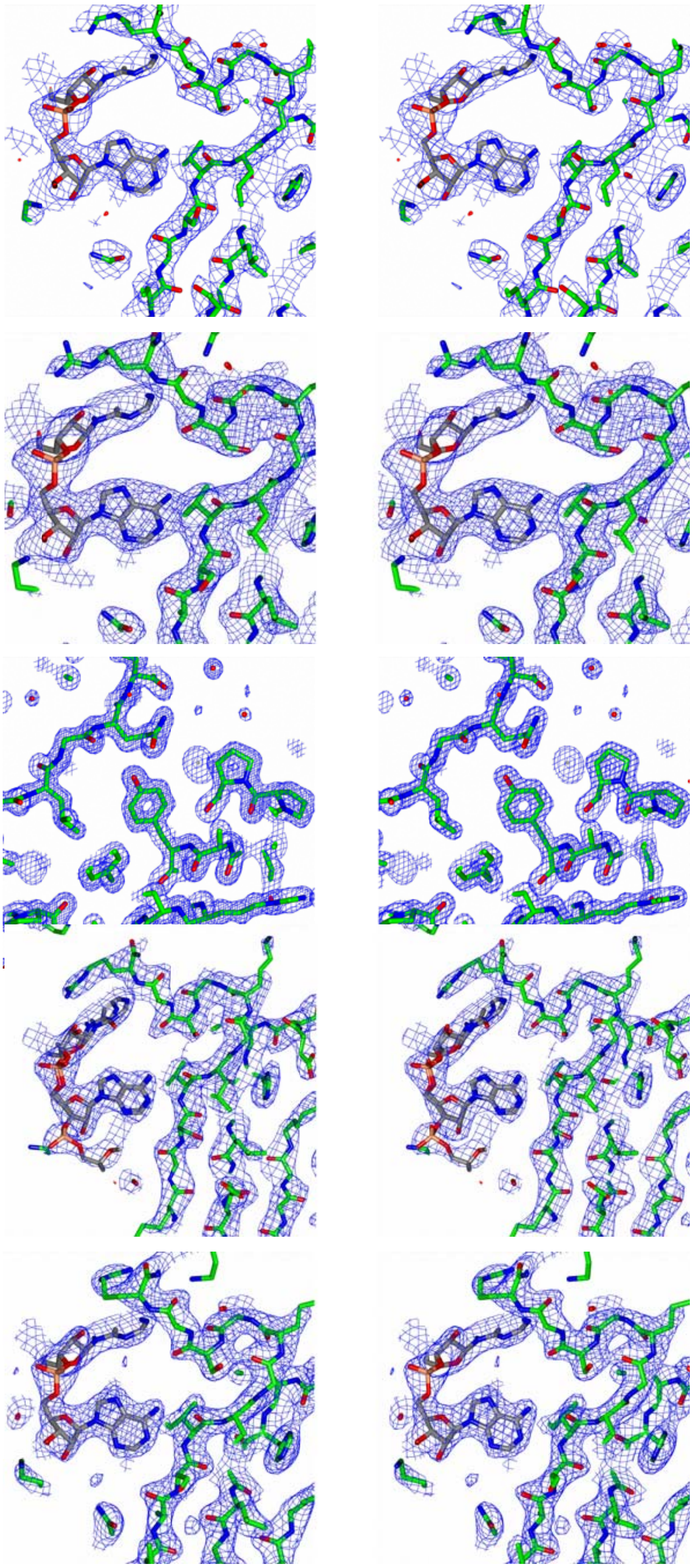

**Supplementary Figure 8: Representative electron density maps of T-STAR structures.**

Representative stereo image of 2Fo-Fc maps contoured at 1.5σ (blue mesh).

Uncropped gel corresponding to Figure 5a (CD44 exon v5)

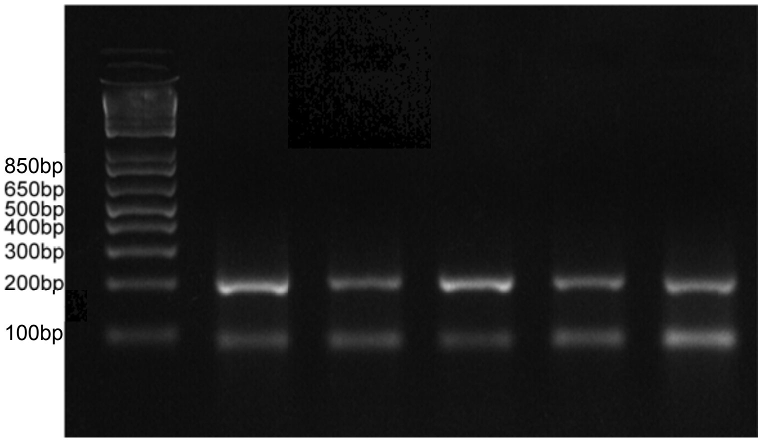


Uncropped gel corresponding to Figure 5b (Neurexin 3 exon AS4)

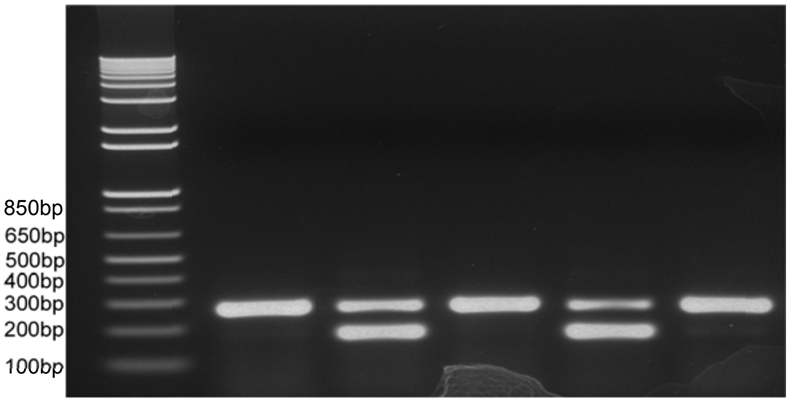


Uncropped gel corresponding to Figure 5c (Neurexin 2 exon AS4)

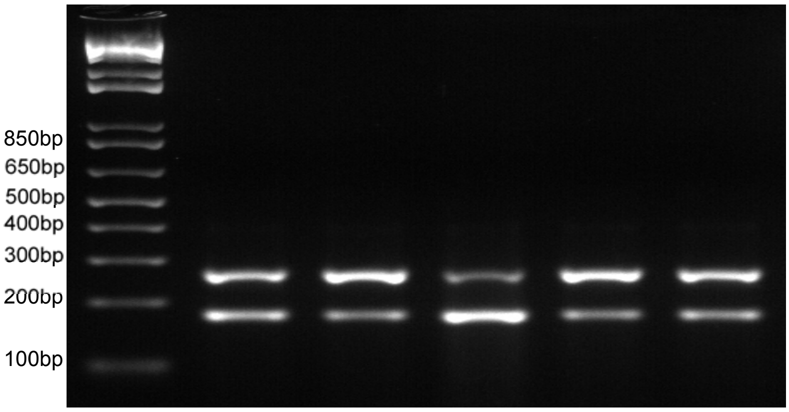


**Supplementary Figure 9: Uncropped gels corresponding to Figure 5 a-c**

## Supplementary Table 1: SAXS analysis of T-STAR and Sam68

| | $R_g$ [Å] | $D_{max}$ [Å] | $V_{Porod}$ [Å$^3$] | $MW_{SAXS}$ [kDa] (dimer) | $MW_{calc}$ [kDa] (dimer) |
|---|---|---|---|---|---|
| T-STAR QUA1-KH | 26 | 87 | 62612 | 39 | 36.9 |
| T-STAR STAR | 28 | 97 | 71472 | 45 | 42.0 |
| Sam68 QUA1-KH | 27 | 96 | 61847 | 38 | 37.5 |
| Sam68 STAR | 30 | 104 | 77673 | 48 | 42.7 |
| Sam68 QUA1-KH AAAUAA | 27 | 90 | 58971 | (37)* | 41.8 |
| Sam68 STAR AAAUAA | 27 | 102 | 71836 | (42)* | 46.9 |

* The density of the RNA is not considered.

**Supplementary Table 2: Most enriched 6-mer motifs bound by T-STAR identified by HITS-CLIP**

| k-mer sequence | k-mer frequency in CLIP tags | k-mer frequency in genomic background tags | Corrected frequency | p-value |
|---|---|---|---|---|
| AAUUAA | 1.034314447 | 0.21112411 | 0.823190337 | < 1.0E-300 |
| AUAAAC | 0.755398192 | 0.199502599 | 0.555895592 | < 1.0E-300 |
| UUAAUU | 0.774767376 | 0.224682539 | 0.550084837 | 4.60E-195 |
| AAUAAU | 0.751524355 | 0.222745621 | 0.528778734 | < 1.0E-300 |
| AUUAAA | 1.104043511 | 0.331213053 | 0.772830458 | 8.14E-197 |
| AAAUAA | 1.456562667 | 0.437743567 | 1.0188191 | 3.00E-238 |
| UAAAAC | 0.768956621 | 0.242114805 | 0.526841816 | 4.57E-246 |
| UAAAUA | 0.842559522 | 0.271168582 | 0.57139094 | < 1.0E-300 |
| AAAAAA | 2.763982614 | 0.923910096 | 1.840072518 | < 1.0E-300 |
| AAAAUA | 1.278366171 | 0.443554323 | 0.834811848 | 9.28E-150 |
| UUAAAU | 0.803821153 | 0.284727011 | 0.519094142 | 0.00E-01 |
| AAAAAU | 1.417824298 | 0.50940955 | 0.908414748 | 2.64E-104 |
| AAUAAA | 1.41588738 | 0.511346468 | 0.904540912 | 7.56E-119 |
| AUAAAA | 1.123412695 | 0.420311302 | 0.703101394 | 4.06E-239 |
| AUAAAU | 0.784451968 | 0.29828544 | 0.486166529 | < 1.0E-300 |

**Supplementary Table 3: Fluorescence polarization affinity measurements of T-STAR and Sam68 QUA1-KH domains to 5-mer RNAs.**

| Kd (µM) | T-STAR QUA1-KH | Sam68 QUA1-KH |
|---|---|---|
| **AUAAA** | 37 | 55 |
| **U**UAAA | 16 | 50 |
| **C**UAAA | 18 | 34 |
| A**A**AAA | 6 | 13 |
| A**C**AAA | 55 | >100 |
| AU**U**AA | >100 | >100 |
| AU**C**AA | >100 | >100 |
| AUA**U**A | >100 | >100 |
| AUA**C**A | >100 | >100 |
| AUAA**U** | 45 | 49 |
| AUAA**C** | 47 | >100 |
| CCCCC | >100 | >100 |
| UUUUU | >100 | >100 |

**Supplementary Table 4: list of primers used in this study**

**Sam68:**

STAR construct:

Forward: 5’-TACTTCCAATCCATGATGGAGCCAGAGAACAAGTACCTG-3’

Reverse: 5’-TATCCACCTTTACTGTCAACCACGAGAGGGTTCAGGTAC-3’

QUA1-KH construct:

Forward: 5’-TACTTCCAATCCATGATGGAGCCAGAGAACAAGTACCTG-3’

Reverse: 5’-TATCCACCTTTACTGTCACATCATATCCGGTACTAG-3’

Y241E mutant:

Forward: 5’-GTCTTTGGACCCCCATGTGAGGCT**GAA**GCTCTTATGGCCCATGCCATGGAG-3’

Reverse: 5’-CTCCATGGCATGGGCCATAAGAGC**TTC**AGCCTCACATGGGGGTCCAAAGAC-3’

**T-STAR:**

STAR construct:

Forward: 5’-TACTTCCAATCCATGATGGAGGAGAAGTACCTGCCC-3’

Reverse: 5’-TATCCACCTTTACTGTCAAACATCTGCATTTTCTGAACC-3’

QUA1-KH construct:

Forward: 5’-TACTTCCAATCCATGATGGAGGAGAAGTACCTGCCC-3’

Reverse: 5’-TATCCACCTTTACTGTCAATTATAATCAGGGATGAGGAACTT-3’

KH-QUA2 construct:

Forward: 5’-TACTTCCAATCCATGATTAATAAGAACATGAAGCTGGGA-3’

Reverse: 5’-TATCCACCTTTACTGTCAAACATCTGCATTTTCTGAACC-3’

KH construct:

Forward: 5’-TACTTCCAATCCATGATTAATAAGAACATGAAGCTGGGA-3’

Reverse: 5’-TATCCACCTTTACTGTCAATTATAATCAGGGATGAGGAACTT-3’

Y141E mutant:

Forward: 5’-GTGTTTGCCCCACCTGCAGAAGCT**GAA**GCCAGGATGGGACATGCTTTGGAA-3’

Reverse: 5’- TTCCAAAGCATGTCCCATCCTGGC**TTC**AGCTTCTGCAGGTGGGGCAAACAC-3’
